# How to Design a Targeted Agricultural Subsidy System: Efficiency or Equity?

**DOI:** 10.1371/journal.pone.0041225

**Published:** 2012-08-02

**Authors:** Rong-Gang Cong, Mark Brady

**Affiliations:** 1 Centre for Environmental and Climate Research (CEC), Lund University, Lund, Sweden; 2 AgriFood Economics Centre, Department of Economics, Swedish University of Agricultural Sciences, Lund, Sweden; University of Minnesota, United States of America

## Abstract

In this paper we appraise current agricultural subsidy policy in the EU. Several sources of its inefficiency are identified: it is inefficient for supporting farmers’ incomes or guaranteeing food security, and irrational transfer payments decoupled from actual performance that may be negative for environmental protection, social cohesion, etc. Based on a simplified economic model, we prove that there is “reverse redistribution” in the current tax-subsidy system, which cannot be avoided. To find a possible way to distribute subsidies more efficiently and equitably, several alternative subsidy systems (the pure loan, the harvest tax and the income contingent loan) are presented and examined.

## Introduction

Payments to farmers are a central part of the EU’s Common Agricultural Policy (CAP) which dictates agricultural policy in all 27 member states. It accounts for almost half of the EU’s budget and almost half of the legislation [1]. Initially the objectives of CAP were to (Rome Treaty in 1955): (1) increase agricultural productivity; (2) ensure a fair standard of living for those engaged in agriculture; (3) stabilize agricultural markets; (4) assure the availability of food; and (5) ensure reasonable prices for consumers.

**Figure 1:**
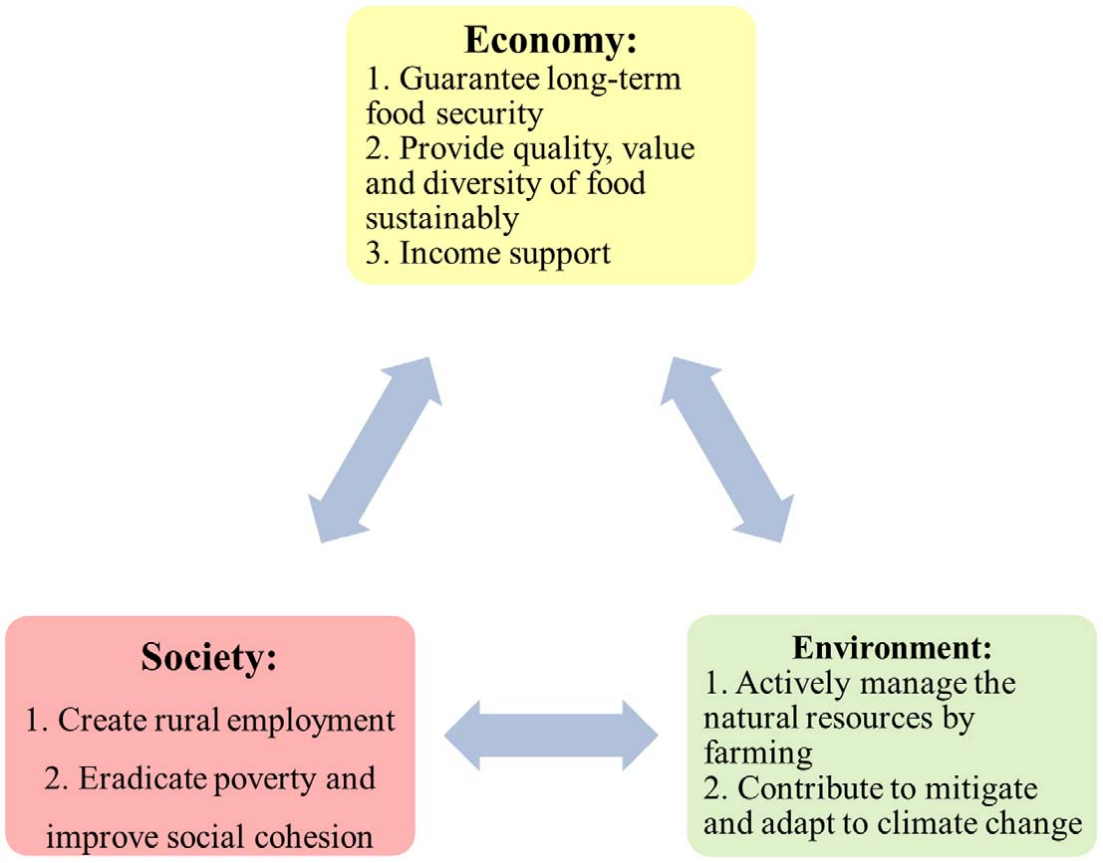
The objectives of CAP from the perspective of Economy, Environment and Society (EES).

In recent years, the role of CAP has been further broadened to (Article 4 of Council Regulation (EC) No 1698/2005): (1) provide high-quality food and non-food products; (2) protect the environment; and (3) promote the harmonious development of different regions. The objectives of CAP can be understood from the perspectives of Economy, Environment and Society (EES), as shown in Figure 1.

The most important instrument of CAP is the Single Payment Scheme (SPS) which is not related to the volume of commodity output; so called decoupled payments. To qualify for subsidies (i.e. payments in the language of the European Commision), farmers are required to keep their land in “Good Agricultural and Environmental Condition” (GAEC) and respect relevant statutory management requirements, together referred to as cross-compliance.

The SPS alone accounts for almost 75% of the CAP budget (€54 billion annually) or 32% of the total EU budget [2]. Member States (MS) were given some freedom to choose how to implement the SPS in 2005. They could choose a regionalized payment with farmers receiving an identical payment per hectare within a region (regional model), a farm-specific payment which is based on each farm’s historical production level (historical model) or a combination of both (hybrid model).

The main advantages of SPS are that farmers’ output decisions are now guided by consumer demand and not distorted by output subsidies, and its benefits for the environment. However, due to the heterogeneity of agricultural and socio-economic conditions in the EU, SPS may also have some disadvantages as follows:

1. The SPS has limited potential for supporting farmers’ incomes which is the original motivation of the support [3]. Current support is highly concentrated to a few large farms, whereas many small farms that are more dependent on support receive only a relatively small share of the total payment.
2. The SPS’s contribution to food security is not as large as imagined because the bulk of the payments are paid to the most fertile regions where market prices are sufficient to guarantee food production [1].
3. SPS has the tendency to be distributed to richer regions and farmers, which may be harmful to social cohesion [4].
4. In practice, there is inadequate feedback between levels of public goods provided by agriculture and payments received by individual farms. Farmers are usually remunerated for carrying out particular management tasks rather than being rewarded directly for measured environmental performance, and payment levels are not related to actual costs.

Future EU agricultural policy should aim to enhance the overall competitiveness of agriculture, protect the environment and promote rural development. However, in general SPS has weak rationale in terms of environmental externalities and social cohesion. So the question as to how to distribute payments reasonably, i.e. to maximize social welfare in terms of the stated goals, is an important issue to study.

The underlying motivation for the current distribution of SPS payments is compensation for historical reductions in agricultural price support (first in 1992 as a result of the MacSharry Reform). One possible justification for SPS could be capital market imperfections which prevent farmers from borrowing for financing investment [5]. Another justification is environmental externalities which cannot be reflected in the market prices [6].

In regard to income redistribution, the current system also has some severe drawbacks. First, farmers with the highest yielding land and hence who are competitive in the market, receive the highest payments per ha. Accordingly, farmers with less fertile land receive lower payments per ha, and hence their farms face marginalization and abandonment, which could have irreversible and detrimental impact on European agricultural production and its cultural landscapes [7]. Secondly, since payments are based on area, the largest farms receive the largest total payments. Consequently, the current CAP and its SPS may imply reverse redistribution, i.e. redistribution from the poor to the rich [8].

In summary, SPS should be better targeted to poorer farmers (small farms) and the environment. In principle, high-income households should receive a low subsidy (if at all) and low-income households a high subsidy [9]. As public goods, environmental products should be subsidized by CAP to cover relevant costs.

However, reality may be more complex. On the one hand, more direct payments to marginal regions and poorer farmers will affect investment and not be conducive to economies of scale [10]. On the other hand, the current subsidy system is hurting small farms and poor farmers through reverse distribution, which will be harmful for social cohesion and environmental protection [11]. Therefore, there is a tradeoff between efficiency and equity.

While the 2003 reform may be the most radical reform of the CAP to date, the concept of SPS or decoupled agricultural support is not new. Decoupling was first proposed more than 50 years ago. Beard and Swinbank provided a comprehensive review of the early proposals to decouple agricultural support in the US in the 1950s and in Europe in the 1960s [12]. Josling further argued that a direct income payment unrelated to output should be a way of ensuring reasonable standards of living for rural people [13].

However, decoupled payments as a form of government intervention have been roundly criticized from different perspectives since its inception. Criticism has been wide-ranging, and even the European Commission has long been persuaded of the numerous defects of decoupled payments. The main opposing viewpoints include:

### (1) Production Distortion

While the payments under SPS may be decoupled from production, they are still a source of income for the farm households and may indirectly affect production decisions through the “wealth effect”. Hennessy studied the relationships between decoupled payments, farmers’ risk preferences and production decisions [14]. He found that if farmers’ risk aversions declined as incomes increased, an increase in wealth as a consequence of the decoupled payment could induce them to take riskier production decisions, and thus increase outputs compared with the situation in which no decoupled payment was made.

Decoupled payments also relax the individuals’ capital constraints thus lowering the cost of capital [15]. Revell and Oglethorpe also suggested the possibility that decoupled payments could affect production through an expectations effect [16]. They claimed that producers might adopt a ‘safety first’ strategy and make only minimal changes to production plans in case future payments were reassessed and again related to production or agricultural activity. Therefore, one of the decoupling’s objectives, not to distort production, is not fully fulfilled in the current policy framework.

### (2) Environmental Problems

The cross-compliance effect is the other objective of decoupled payments. However, some empirical results show that this effect is also complicated. Based on two micro-economic models (AgriPolis and MODAM), Uthe et al. found that in the case of grassland, decoupling led to improvement of the environment as a result of the cross-compliance obligations [17]. However, with respect to arable land, decoupling led to negative environmental effects due to changes in the crop mix, with less cereals and a greater area of more intensive winter rape and row crops being grown.

### (3) Hurting Small Farms

Although most policy makers in Europe agree that they want to promote "family farms" and small scale production, decoupled payments in fact benefit large farms much more than small farms, because decoupled payments are linked to farm size. So while subsidies allow small farms to persist, large farms tend to receive the greatest share of the subsidies. Within the 2008 Health Check of the CAP [18], a first step was taken to limit decoupled payments to very large landowners.

There are also some other criticisms such as the equity among member states [19], the unfair competition with developing countries [20] and so on. Among them, the tradeoff between efficiency and equity is the source of many controversies.

Motivated by the experiences of EU and the United States, we attempt to construct a simplified economic model and answer the following questions:

1. How can we define efficiency and equity in regard to agricultural policy? What are the underlying conflicts between efficiency and equity in the current SPS?
2. Is there an integrated subsidy system that can achieve a balance between efficiency and equity?

The results may shed light on the wisdom of the current CAP and the proposed 2013 reform. Also, some conclusions regarding general rules for designing agricultural subsidy systems are provided. The article is structured as follows: firstly, a theoretical analysis of efficiency and equity in the current SPS is presented; secondly, we compare the efficiency and equity of three novel options for agricultural subsidies; finally, conclusions and policy recommendations according to the current direction of CAP reform are given.

## Analysis

### 1. Models

The model presented in this paper is inspired by the work of García-Peñalosa and Wälde [8]. In this section, a small open economy and relevant assumptions are initially described. Next, the perfect market and its operating mechanism are presented for reference. The main variables and functions are listed in Appendix S1.

#### 1.1 Description of the economy

A small open economy with a population of constant size *N* is considered here. All individuals live for two periods and are identical in all respects except for their initial wealth which is denoted by *n*. Its frequency distribution is given by *f* (*n*). At the beginning of the first period, people choose whether to work in the agricultural sector or in other sectors. If people choose to work in other sectors, they will receive an income in both periods. Otherwise they enroll in the agricultural sector. In this case, they do some farming in the first period and harvest (realize their income) in the next. If they choose to work in the agricultural sector, they also need to decide whether to produce only agricultural products or to produce both agricultural and environmental products (e.g. landscape). In this paper, farmers are assumed to produce both agricultural and environmental products because the latter is currently profitable.

Farmers are categorized based on the sizes of their farms. It is assumed that there are two sizes of farms based on the area of agricultural land: small farms (*s*) and large farms (*l*). The exogenous rental prices for these are *r_s_* and *r_l_* respectively, where *r_s_* < *r_l_*. Economies of scale and heterogeneous soil fertility are not considered in this paper.

**Figure 2:**
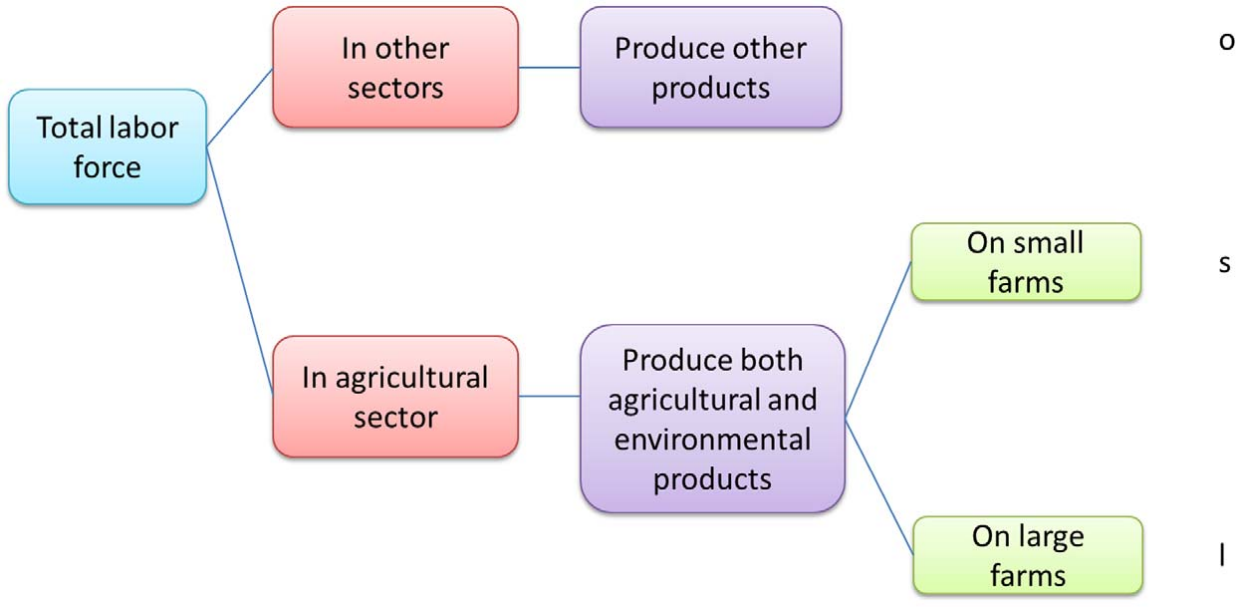
The structure of the labor force.

Consequently the total labor force can be divided into three categories: (1) Individuals who are employed in other sectors (*o*); (2) Farmers who produce both crops and environmental products on small farms (*s*); and (3) Farmers who produce both crops and environmental products on large farms (*l*). The three types of labor (*o*, *s* and *l*) are illustrated in Figure 2. Given that 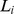 (*i* = *o*, *s* or *l*) is the size of the labor force of each type *i*, then 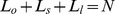.

The costs of the different types of labor are given in Table 1. For individuals in other sectors, they don’t need to invest any money for their employment. For individuals in the agricultural sector, they need to pay the rent for the farm’s land (*r_s_* or *r_l_*) and also the costs of generating environmental products in period one (i.e. prior to receiving any income).

**Table 1:** Costs of different types of labor.

	Land rental costs		
Type of labor	Small farm	Large farm	Costs of producing environmental products
*o*			
*s*	√		√
*l*		√	√

It is assumed that borrowing in order to finance costs for individuals in the agricultural sector is not possible in reality as agriculture is high risk and not satisfactory collateral for private lenders. Hence, in the absence of government intervention, an individual can only enroll in the agricultural sector if his (her) initial wealth (*n*) is large enough to cover his (her) costs.

The economy produces three types of products: agricultural products (*A*), environmental products (*E*) and other products (*O*). Since we desire to study individuals’ career choices, we omit other production factors and only focus on the amount of labor. Define *I*(), *AL*(), *AH*() and *G*() as the production functions for other products, agricultural products from small and large farms, and environmental products respectively as shown in equations (1)–(3).


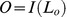
(1)


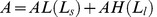
(2)


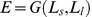
(3)

The purpose of introducing production functions is to determine returns to labor in the different sectors. In a perfect market, returns should be equalized across all sectors. Otherwise, individuals in the low income sectors will move to the high income sectors. In this paper, other endowment differences between individuals, such as abilities, are not considered. Consequently, for a given total population, there is an optimal labor structure for which there is a maximum total income for the entire population.

More specifically, as a production factor, the marginal output of labor should be positive but show a decreasing trend when the amount of labor is increasing. Therefore, first derivatives of production functions are positive while second derivatives are negative. In a small open economy, 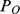, 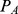 and 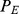 are prices for other products, agricultural products and environmental products respectively which are determined exogenously. The return (*Py*) of each labor force is the product of its marginal output and corresponding price, as shown in equations (4)–(7):


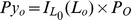
(4)


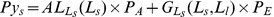
(5)


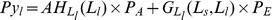
(6)


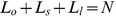
(7)

where equation (7) is the population constraint.

The role of the government is to buy environmental products, subsidize farmers if necessary and levy taxes to maintain a balanced budget. We will examine the characteristics of several possible subsidy systems below.

#### 1.2 The benchmark case of perfect capital markets

We assume a situation where capital markets are perfect as our benchmark. All people can borrow and there is no need for government intervention. Neither taxes and subsidies, nor uncertainty and risk aversion are considered in this section. The only role of government in the perfect capital market is to set the price of environmental products and buy them. The lifetime income (*W*) of an individual who is employed in other sectors is the present value of payments from other products for two periods, as shown in equation (8).


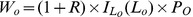
(8)

where *R* is the exogenous discount rate.

The lifetime income of an individual in the agricultural sector is equal to the sum of the discounted revenues from agricultural and environmental products minus the land rent (*r*) and the cost of producing environmental products (EC) which are payable in the first period, as shown in equations (9)–(10).


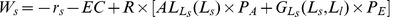
(9)


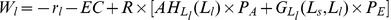
(10)

The cost for producing environmental products, EC, is fixed and identical for both farm types.

Since there are not any barriers for employment, individuals with lower incomes can move to the high-income sector, which would reduce the high-income sector’s returns to labor and boost those in the low-income sector. Consequently, in an equilibrium economy, all individuals’ incomes will be equalized (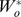 = 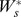 = 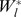). Equations (8)–(10) are simultaneously solved to obtain the optimal structure of labor 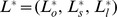, which is shown in equation (11).


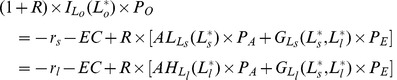
(11)

**Figure 3:**
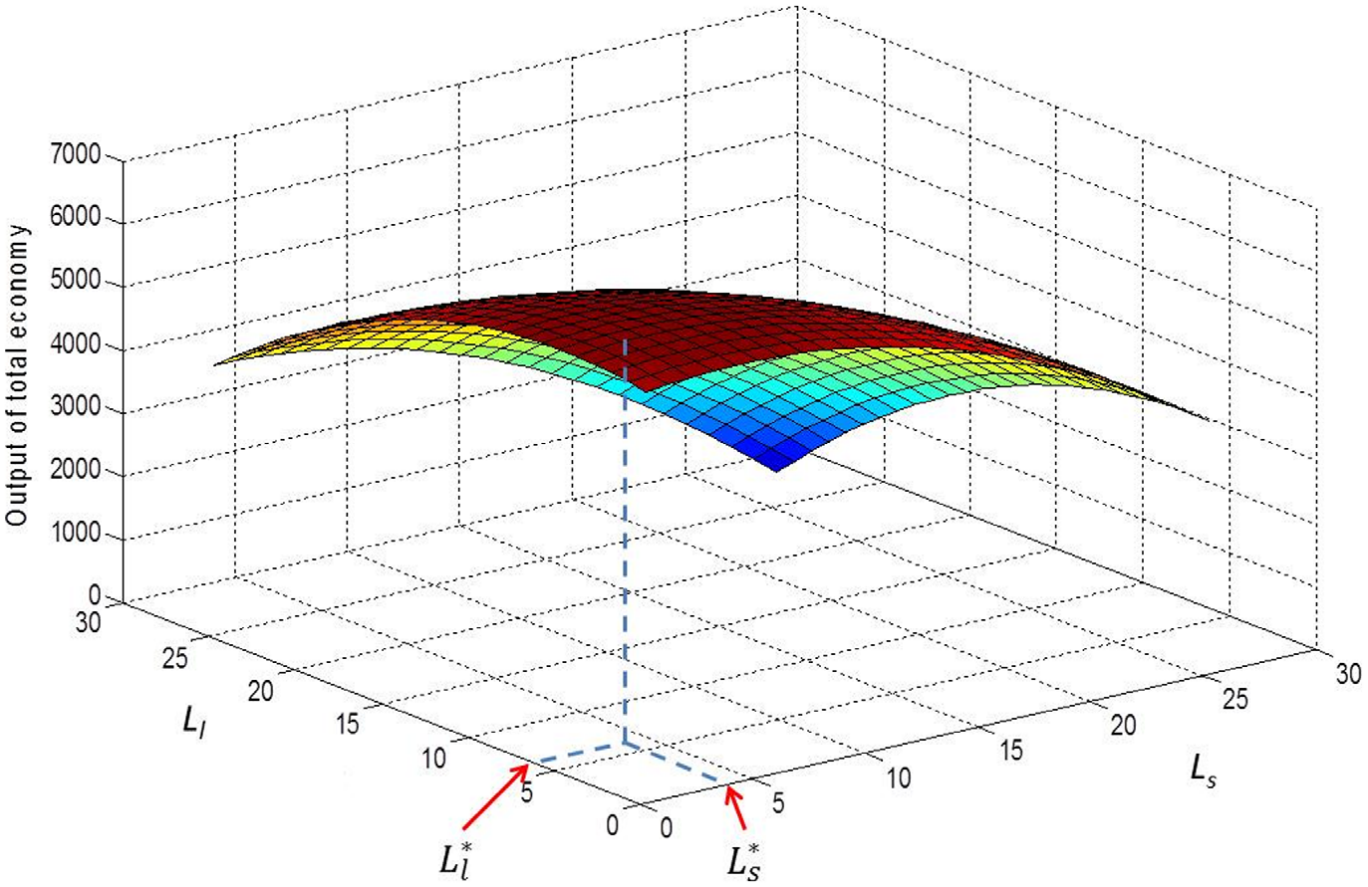
The efficient size of the agricultural work force with small and large farms when borrowing is possible. Note to Figure 3: Although we have three types of labor (*s*, *l* and *o*), the amount of the third type of labor, other sectors, is determined by the amounts of the other two types of labor because the total population is fixed, i.e. 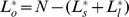.

The optimal labor structure depends on the factor prices (*r_s_*, *r_l_* and EC) and the product prices (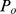,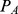 and 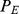). The labor structure 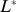 is also called the efficient level of labor and will be our point of reference (as shown in Figure 3).

In the next section, the implications of three subsidy systems under imperfect markets are analyzed. They are the current SPS system, subsidies to achieve the efficient level of labor in each sector (i.e. that maximizes output as shown in Figure 3) and subsidies to achieve the equitable level of lifetime income (all individuals have an equal lifetime income).

### 2. The Working of Traditionally used Tax-subsidy System

In reality, farmers might have difficulty obtaining loans to finance their costs for renting land and producing environmental products as shown in Section 1.2. Therefore, a subsidy is needed to aid poor farmers while a tax is collected from the entire population to keep a balanced budget. The aim of this section is to examine the current SPS and determine the optimal labor structure in the context of the chosen subsidy and taxation policy (Section 2.1). Also two specific examples of the SPS (subsidizing the efficient level of labor and the equitable level of lifetime income) are discussed.

#### 2.1 The current SPS system

We assume that *T* is a lump-sum tax levied on all individuals in the first period of their lives. In the current SPS system, every farmer who produces certain environmental products is eligible for the subsidy. Small farms receive subsidy *S1*, while large farms receive *S2*. In practice, SPS provides subsidies according to the size of the farms. Therefore, in general there is a relationship that 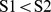.

The government chooses the subsidy rate and then sets the lump-sum tax so as to maintain a balanced budget, as shown in equation (12), which means that the required level of tax is equal to total subsidies.


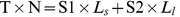
(12)

Individuals in other sectors (*o*) pay a tax but receive no subsidy. Farmers (*s* and *l*) pay the same tax but receive different subsidies. This implies a net transfer to farmers because there are more individuals paying the tax than receiving the subsidy (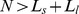).

Define 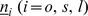 as the minimum level of initial wealth in order to be able to cover *i*’s costs. In the absence of borrowing, an individual’s initial wealth must be large enough to cover the subsidized costs plus the tax. For example, for farmers with small farms (*s*), their required minimum initial wealth, 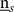, can be calculated based on equation (12), as shown in equation (13).


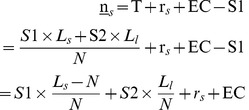
(13)

For farmers with large farms (*l*), their required minimum initial wealth, 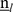, can be calculated as shown in equation (14).


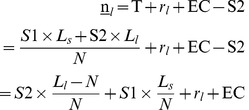
(14)

It is assumed that 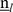 is larger than 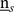 because farmers with large farms need more investment (i.e. pay higher land rents). Clearly, the higher the subsidy, the lower the level of initial wealth required for being in the agriculture sector. For clarity, it is assumed that the labor structure under the current SPS is 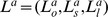. Suppose that under the subsidies of *S1* and *S2*, the capital market constraint is still binding for some individuals, so that more individuals want to be in the agriculture sector than can afford to be (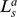
<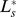,
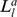
<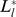), which means that the lifetime income of farmers may exceed the lifetime income of people in other sectors. In this case, the number of individuals having small (large) farms is equal to the number of individuals whose initial wealth is between 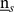 and 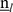 (larger than 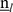) as shown in equations (15) and (16).


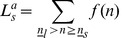
(15)


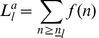
(16)

Equation (15) and (16) can be used with equations (13) and (14) to jointly determine the sizes of *s* and *l,* and the minimum initial wealth levels required for entering the agricultural sector (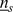 and 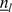), as a function of the subsidies *S1* and *S2*, and the distribution of initial wealth 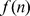.

#### 2.2 Subsidizing the efficient level of labor

Suppose that the purpose of the government is to maximize the economic output of a given generation; then they should design a subsidy system to achieve the optimal labor structure 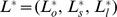. In this case, there should be exactly 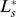 individuals whose initial wealth is between 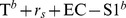 and 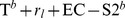, and 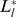 individuals whose initial wealth is larger than 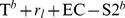 where 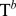, 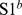 and 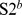 are the tax, subsidy for farmers with small farms and subsidy for farmers with large farms to achieve the optimal labor structure respectively.

**Figure 4:**
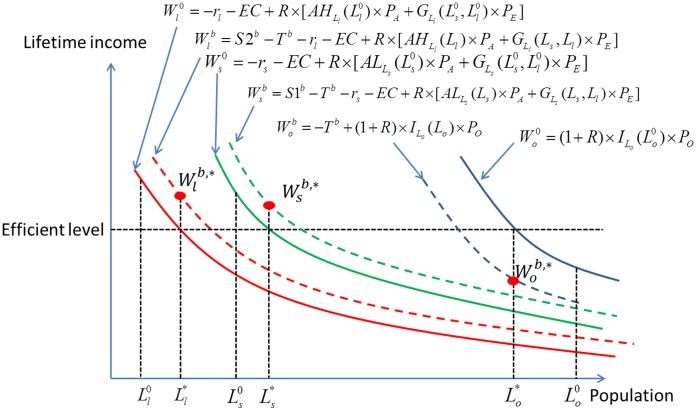
The efficient subsidy rate.

Figure 4 examines graphically the effects of the subsidies 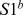 and 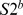 on the lifetime incomes of the three types of labor. The solid lines represent lifetime incomes in the absence of subsidies, where 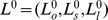 is the labor structure in this case. 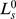 and 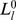, which are numbers of *s* and *l* whose initial wealth can afford agricultural costs, are smaller than 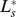 and 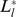 respectively. At 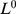, lifetime incomes are higher for *s* and *l* but lower for *o* than under perfect markets. Under the tax-subsidy system (subsidizing the efficient level), the lifetime incomes of *o* are the incomes received in two periods minus the tax, i.e. 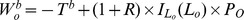. Hence, the introduction of the tax represents a downward shift of the curve 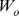. The lifetime incomes of *s* and *l* are 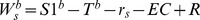
×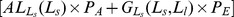 and 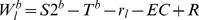
×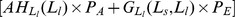 respectively. Because *S1^b^* and *S2^b^* are larger than T^b^, the lifetime incomes of *s* and *l* increase, which means upward shifts of 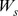 and 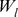. The subsidies can then be set to 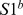 and 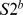 so that the distribution of labor is exactly 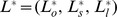, as with perfect capital markets.

The efficient subsidies (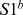 and 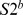) have two distributional consequences:

Firstly, all individuals are paying taxes that are distributed only among those farmers with higher incomes, implying that there is a transfer of resources from the poor to rich individuals. This is what is called “reverse redistribution”.

Secondly, the introduction of efficient subsidies leads to a situation where those who have large farms enjoy larger incomes. The efficient subsidy does not remove inequality. And it also fails to provide an equality of chances. The difference in the lifetime incomes of individuals with large (small) farms and individuals in other sectors is 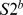 (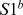). Therefore, there is reverse redistribution under the SPS scheme, not only from the non-agricultural sectors to the agricultural sector, but also from all farmers to farmers with large farms.

The efficient subsidies not only fail to equalize life-time incomes, but also fail to provide ex ante equality of chances. Even though some relatively poor individuals can now afford to enter the agricultural sector, the greatest opportunity is still offered to the richest individuals. As a result, poorer individuals are still systematically excluded from agriculture.

#### 2.3 Subsidizing the equitable level of lifetime income

In this section, subsidies which can guarantee the equality of lifetime incomes are examined. The labor structure in this case is termed the equitable labor structure, 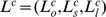. The equitable subsidies (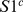 and 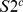) and their corresponding tax (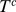) are defined by equation (17).


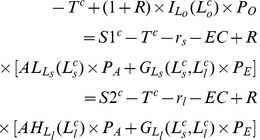
(17)

What then is the equitable labor structure 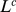
? In the section above, it is known that for the efficient labor structure, 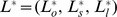, the lifetime incomes of *s* and *l* are larger than that of *o*. In order to reduce the incomes of the former, the subsidies should be further increased to increase the numbers of *s* and *l*, thus reducing farmers’ incomes and raising incomes in other sectors. Therefore, 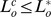, 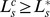 and 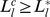 are all satisfied.

The increases in the numbers of *s* and *l* imply that their marginal products are lower than their marginal costs. Therefore, the equitable labor structure, 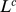, is not efficient since too many people enter the agricultural sector.

Consequently the SPS, as a form of tax-subsidy system, is characterized by a trade-off between efficiency and equity: efficient subsidies imply inequality in lifetime incomes; while equitable subsidies induce an excessively large number of farmers and thus reduction in the total value of economic output. Despite the equality of lifetime income, there is still not equality of opportunity, as those with a very low level of initial wealth will still not be able to afford agricultural costs. The only way around this is to provide a subsidy that covers full costs; but this will further increase the efficiency loss.

### 3. Some Possible Solutions–the Pure Loan, the Harvest Tax or the Income Contingent Loan?

As the previous discussion has shown, SPS as a traditional tax-subsidy system that implies reverse distribution cannot achieve the targets of efficiency and equity simultaneously. Therefore, it raises the question whether there is a better solution for an agricultural subsidy system. Firstly, the pure loan system is presented; secondly, to solve the problems caused by uncertainty and risk-aversion, the harvest tax and the income contingent loan systems are presented. Finally, some possible policy options for EU in the future are given for reference.

#### 3.1 The pure loan scheme

A straightforward solution, which removes the constraints imposed by imperfect capital markets without generating reverse redistribution, is to abolish all subsidies and introduce a government loan system. Agricultural costs would be fully financed, and the capital market imperfections would be overcome by loans provided by the government. In our highly stylized economy, all individuals would then have identical lifetime incomes, the allocation of resources would be efficient, and any individuals would have the opportunity of entering into agriculture.

However, the pure loan scheme neglects an important aspect which we have so far not taken into account: the risks related to agricultural production. Agricultural production is a risky investment, particularly risk stemming from variations in the weather. Hence, from the farmers’ perspectives, there is risk associated with farming. A simple form of uncertainty is presented in what follows assuming the possibility of successful agricultural production is set exogenously.

It is supposed that farmers, irrespective of farm size, will harvest in the second period with probability *p* (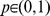) and fail to harvest with probability 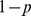. If farmers fail, they will enter other sectors and receive a salary in the second period. Individuals are assumed to be risk averse and have utility functions denoted by 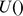. Assume that the expected returns of farmers with large farms are larger than those of farmers with small farms which are in turn larger than those of individuals in other sectors, as shown in equation (18).


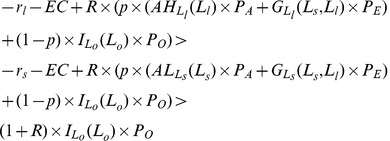
(18)

Different from the analysis in sections 1 and 2, is that the expected returns of farmers are larger than individuals in other sectors due to individuals’ risk aversions, as in reality. If there is no difference in lifetime incomes between farmers and individuals in other sectors, people would prefer the latter to obtain certain incomes.

Under a pure loan system, individuals who work in other sectors have total wealth of 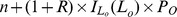; farmers with small farms who succeed have total wealth of 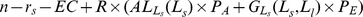; farmers with small farms who fail have total wealth of 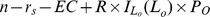; farmers with large farms who succeed have total wealth of 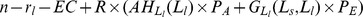; and farmers with large farms who fail have total wealth of 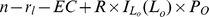. For simplicity, the marginal outputs of production functions are assumed to be constant in the two periods.

Function 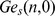 is defined as the difference between the expected utility of farmers with small farms and that of individuals in other sectors in the case of no subsidy, as shown in equation (19) where *n* is the individual’s initial wealth and 0 stands for no subsidy.


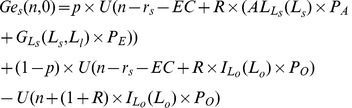
(19)

Function 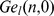 is defined as the difference between the expected utility of farmers with large farms and that of individuals in other sectors in the case of no subsidies, as shown in equation (20).


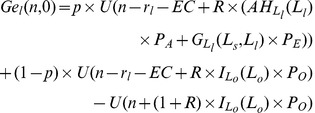
(20)

**Figure 5:**
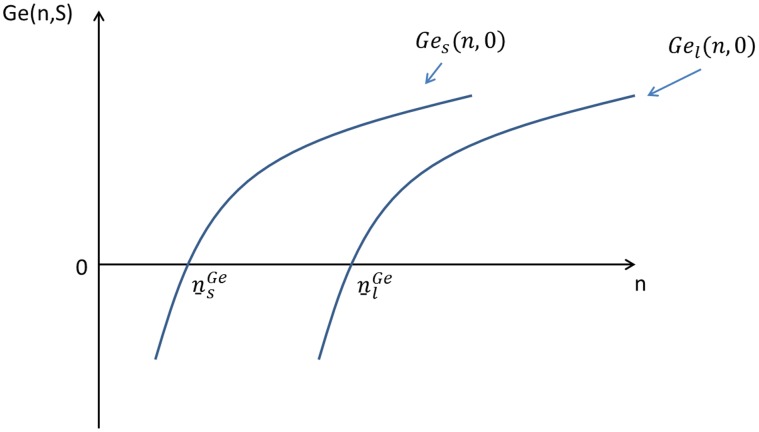
Initial wealth and the willingness to enter agriculture.

When individuals are sufficiently risk averse and their initial wealth, *n*, is small enough, 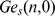 and 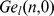 are both negative which implies that poor individuals will be very sensitive to the risks in the agricultural sector when income represents a large proportion of their wealth. However, when their initial wealth, *n*, is relatively large, 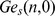 and 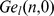 will be positive, implying that rich individuals will be more willing to enter the agricultural sector. Define 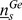 and 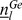 as threshold levels where 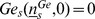 and 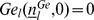, as shown in Figure 5. Individuals, whose initial wealth is larger than 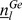, will invest in large farms (
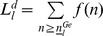
); while individuals whose initial wealth is between 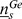 and 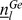 will invest in small farms (
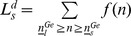
). Remaining individuals will enter the other sectors.

If there is no uncertainty, i.e. the case of equation (18), the socially optimal labor structure is 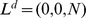. Therefore, when there are uncertainty and risk aversion, the pure loan scheme won’t result in an efficient allocation. It is also not equitable due to ex post differences between the lifetime incomes of different types of labor, as shown in equation (18). Finally there is no equality of chance. Although all individuals can get loans to cover the costs of agriculture, only rich individuals will choose to invest in agriculture because they can afford to take the risk.

#### 3.2 The harvest tax system

The harvest tax system, as defined here, has two components. Firstly, there is a public loan scheme, so that any individual can obtain a loan that has to be fully paid back. In addition to making loans available, the government can finance part of the agricultural costs through a subsidy. The total subsidy is then repaid by levying a tax on those who make a profit from agricultural production. Those who don’t make a profit from agriculture don’t need to pay the harvest tax. Unsuccessful farmers, hence, receive a net subsidy, while successful farmers have to pay back not only their own loans but also the subsidy received by those who fail.

For clarity and simplicity, it is assumed that the government gives the same subsidy 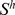 to *s* and *l*. In the second period, only the farmers who succeed pay the tax 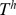. To keep a balanced budget, the following relationship should be satisfied as shown in equation (21).


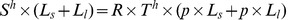
(21)

Farmers who succeed will have an expense for the harvest tax system, as shown in equation (22).


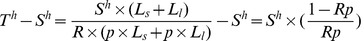
(22)

Farmers who fail will achieve a net income 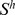 for the harvest tax system. Compared with the pure loan system, the gap between successful and unsuccessful farmers becomes smaller. The variance of farmers’ lifetime incomes however becomes lower, which shows an insurance property of the harvest tax system.

**Figure 6:**
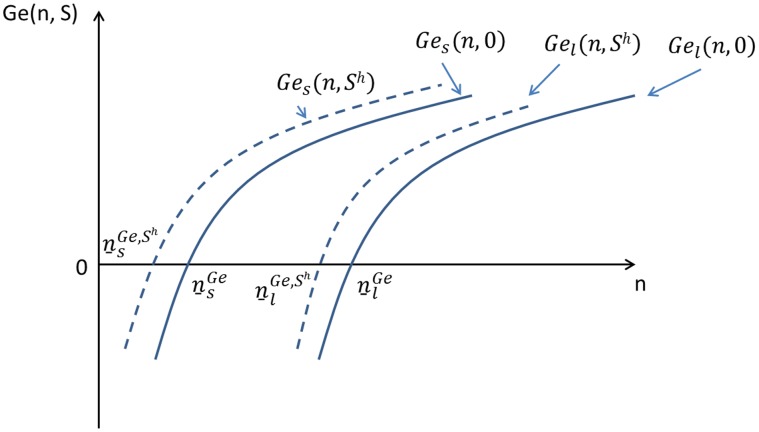
Initial wealth and the willingness to enter agriculture under the harvest tax system.

Under the harvest tax system, function 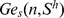 becomes:


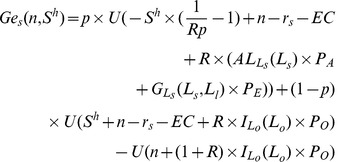
(23)

and function 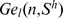 becomes:


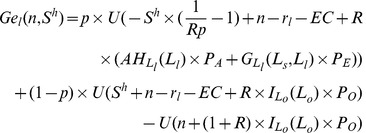
(24)


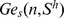
 and 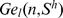 are both functions of subsidy 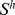. When 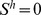, it returns to the pure loan system. For any levels of initial wealth, a higher subsidy 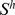 implies a smaller gap (risk) between successful and unsuccessful farmers. An increase in 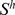 thus shifts 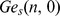 and 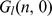 upwards, as shown in Figure 6. For a given subsidy 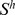, an individual whose initial wealth is larger than 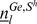 will choose to enter the agricultural sector with a large farm; an individual whose initial wealth is between 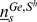 and 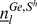 will choose to enter agriculture with a small farm; remaining individuals will choose to work in other sectors. However, the socially optimal labor structure *L^d^* (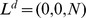) cannot still be achieved as long as 
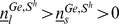
.

The harvest tax has two desirable equity implications: it does not imply reverse redistribution, and it reduces the differences between the ex post lifetime incomes of successful and unsuccessful (i.e. unlucky) farmers. However, there remain differences between individuals as far as their willingness to undertake risk is concerned. Individuals with a large initial wealth are more likely to enter agriculture than poorer individuals. The harvest tax, by providing some degree of insurance, weakens this effect but does not eliminate it. Equality of opportunity is still not achieved.

#### 3.3 The income contingent loans

Another possible policy option is a system of income contingent loans. An income contingent loan is a loan such that: (1) repayment only takes place in the event that an individual’s income exceeds a pre-specified level; (2) annual repayment doesn’t constitute more than a certain proportion of an individual’s income; (3) repayment ceases once the loan plus interest has been repaid [8].

For clarity and simplicity, farmers will borrow 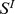 in the first period. The successful farmers will repay their own loans in the second period. To keep a balanced budget, a lump-sum tax 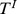 is levied on all individuals to cover the costs of unsuccessful farmers, as shown in equation (25):


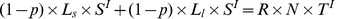
(25)

The difference between the expected utility of farmers with small farms and that of individuals in other sectors, 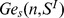, is shown in equation (26):


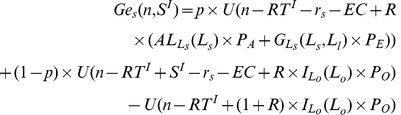
(26)

The difference between the expected utility of farmers with large farms and that of individuals in other sectors, 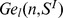, is shown in equation (27):


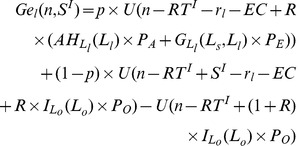
(27)

The difference between expected lifetime income of *s* (*l*) under the income contingent loans and under the harvest tax system is:


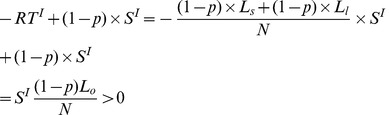
(28)

This reflects the fact that farmers are subsidized by individuals in other sectors. In the income contingent loan system, it is less attractive to enter other sectors compared with the harvest system, which will produce a more efficient labor structure.

The higher expected lifetime incomes of farmers in the income contingent loan system are at the expense of individuals in other sectors who become worse off because they have to pay the tax, 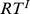. Successful farmers earn as much as under the pure loan system and more than under the harvest tax system. Unsuccessful farmers will earn more than under the pure loan system (as they don’t have to repay their loans but only RT) and either more or less than under the harvest tax system. They earn less if 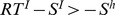 which holds if the subsidy 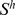 under the harvest tax system is large enough.

Therefore, on the one hand, it is possible with the harvest tax system to outperform a contingent loan system, provided that the subsidy is large enough; on the other hand, the harvest tax system is more equitable as it avoids the reverse distribution to some extent.

In summary, the income contingent loan system is similar to the harvest tax system and therefore is characterized by most of its advantages over the traditional system. In particular, it also provides insurance and results in a more efficient number of farmers. As the maximum repayment under the income contingent loan system is limited to the loans of successful farmers, some general taxes are still needed to subsidize unsuccessful farmers. In contrast to a harvest tax system, it again implies that reverse redistribution occurs. However, it may be more practical to implement because successful farmers don’t have to pay more than their loans.

**Table 2:** Policy options.

Option	No.	Name	Description
1	Reference	Current policy (2010)	Decoupled payments
2	I	Payment based on farmer’s income and a subsidy for environmental products	The subsidy should primarily be distributed to poor farmers.
	II	Dynamic hybrid model (Ref and I)	The combination of reference and I which implies slower reform.
3	III	A pure loan plus a subsidy for environmental products	Farmers can take up a loan and repay it after they harvest. Any individual has the opportunity to enter agriculture.
	IV	An income contingent loan system plus a subsidy for environmental products	A system of income contingent loans makes repayments conditional on whether the income of the farmer exceeds a pre-specified level and computes repayments as a percentage of their earnings. The maximum amount to be paid is the loan plus interests.
	VI	A harvest tax plus a subsidy for environmental products	A harvest tax system makes repayment contingent on income. Repayments from successful farmers exceed the cost of their loans. The difference between repayment and cost is used to subsidize unsuccessful farmers.

#### 3.4 Some policy options for EU in the future

According to the European Commission, there are three main directions for the CAP to take in the future: (1) keep the current direct payment system unchanged; (2) introduce more equity in the distribution of direct payments. Decoupled payments would be composed of a basic rate serving as income support and a compulsory additional payment for specific “greening” public goods; or (3) phase-out decoupled payments in the current form and provide instead limited payments for environmental products. According to the results in this paper, three alternative policy options are given in Table 2.

## Results and Discussion

The motivation of this paper is to prove that the tax-subsidy system currently used to finance the agricultural sector in the EU is characterized by “reverse redistribution”–rich farmers are subsidized by poor farmers and the rest of society. Assuming that government intervention is needed due to the difficulty of obtaining private loans to finance the agricultural sector, some possible policy options that can avoid reverse redistribution are analyzed: the pure loan system, the harvest tax system and the income contingent loan system.

The three systems are identical when agricultural output is certain. When there is agricultural risk, the systems differ in how the subsidy should be financed. Whereas under a pure loan system the farmers repay in full their loans plus interest, a harvest tax system makes the repayments of loan costs contingent on whether the farmers make a profit from agricultural production. Farmers making losses (or not achieving the minimum income threshold) don’t need to repay their loans. Successful farmers, on the other hand, are required to repay their loans plus an extra amount to cover the costs of unsuccessful farmers. In the case of the income contingent loan, it still requires some general taxation to subsidize the less successful farmers and hence has some reverse redistribution effects.

These loan systems may seem unrealistic given the current CAP and the general acceptance of providing farmers with subsidies. The major issues driving CAP reform (section 3.4) seem however to be uncontentious A) there should be more equity in the distribution of agricultural support; and B) farmers should be paid for environmental provisioning. The relevant economic question is therefore the means necessary to achieve these ends. One needs to look no further than the education sector to find examples of loan systems being used in practice to deal with concerns of efficiency and equity [21]–[23]. As our analysis shows, reverse redistribution is unavoidable given the current tax-subsidy basis of the CAP; so the answer cannot be found in the current thinking. However, if payments are offered for environmental products according to demand then a loan system will ensure that sufficient farmers enter or stay in the sector–despite an imperfect capital market–to deliver efficient quantities of these services, e.g. small or low-income farms can finance themselves and repay loans after outcomes.

Since differences in abilities between individuals are not considered in this paper (only differences in initial wealth), an equal distribution of income is also the equitable distribution of income because all individuals will have the same productive capacity. What the solution might be when considering heterogeneity in individuals’ abilities may be another interesting issue. Further the harvest tax system and income contingent loan system presented in this paper are based on the actual incomes obtained by the farmers, hence moral hazard–which implies that farmers may conceal their real incomes to obtain financial advantage–is a relevant issue and a potential subject for future work. Another extension of this paper is to analyze the effect of environmental costs on the desirable distribution of subsidies.

## Supporting Information

Appendix S1 **Main variables and functions used in the paper.**
(DOCX)
