# Supplementary material for: How to Design a Targeted Agricultural Subsidy System: Efficiency or Equity?
Source: PLoS One. 2012 Aug 2;7(8):e41225. doi: 10.1371/journal.pone.0041225 (PMC3410909; doi:10.1371/journal.pone.0041225)
Supplement: Appendix S1 — Main variables and functions used in the paper. (DOCX) [file pone.0041225.s001.docx]

Appendix

Table A1 Main variables used in the paper

| Variable | Definition | Variable | Definition |
| --- | --- | --- | --- |
| *N* | Size of population | *n* | Initial wealth |
| s | Individuals with small farms | o | Individuals in other sectors |
| r_s_ | Land rental for small farms | l | Farmers with large farms |
| L_i_ | Size of labor force of type i (i=*o*, *s* or *l*) | r_l_ | Land rental price for large farms |
| A | Agricultural products | EC | Costs for environmental products |
| O | Other products | E | Environmental products |
| $P_{A}$ | Price of agricultural products | $P_{O}$ | Price of other products |
| *Py* | Wage for labor | $P_{E}$ | Price of environmental products |
| R | Exogenous discount rate | W | Lifetime income |
| T | Lump-sum tax in the current SPS system | $L^{*}$ | Optimal labor structure in a perfect market |
| S2 | Subsidy for farmers with large farms in the current SPS system | S1 | Subsidy for farmers with small farms in the current SPS system |
| $L^{a}$ | Labor structure under current SPS system | $\underline{\text{n}}_{i}$ | The minimum level of initial welfare in order to be able to cover *i*’s costs |
| S1^b^ | Subsidy for farmers with small farms to subsidize the efficient level | T^b^ | The lump-sum tax to subsidize the efficient level |
| W^b^ | The lifetime income (subsidizing the efficient level) | S2^b^ | Subsidy for farmers with large farms to subsidize the efficient level |
| $\underline{n}_{s}^{Ge}$ | Threshold level where $Ge_{s}(\underline{n}_{s}^{Ge},0)=0$ | $L^{c}$ | Equitable labor structure |
| L^d^ | The social optimal labor structure in reality | $\underline{n}_{l}^{Ge}$ | Threshold levels where $Ge_{l}(\underline{n}_{l}^{Ge},0)=0$ |
| $T^{h}$ | Tax in the harvest tax system | $S^{h}$ | Subsidy in the harvest tax system |
| $T^{I}$ | Tax in the income contingent loan system | $S^{I}$ | Subsidy in the income-contingent loan system |

Table A2 Main functions used in the paper

| Function name | Definition | Function name | Definition |
| --- | --- | --- | --- |
| *I*() | Production function for other products | *AL*() | Production function for agricultural products from small farms |
| *AH*() | Production function for agricultural products from large farms | *G*() | Production function for environmental products |
| *f*(*n*) | The frequency distribution of *n* | *U*() | The utility function |
| $Ge_{s}(n,S)$ | The difference between the expected utility of farmers with small farms and that of individuals in other sectors in the case of subsidy S | $Ge_{l}(n,S)$ | The difference between the expected utility of farmers with large farms and that of individuals in other sectors in the case of subsidy S |
